# First draft genome sequence of a strain belonging to the *Zoogloea* genus and its gene expression in situ

**DOI:** 10.1186/s40793-017-0274-y

**Published:** 2017-10-18

**Authors:** Emilie E. L. Muller, Shaman Narayanasamy, Myriam Zeimes, Cédric C. Laczny, Laura A. Lebrun, Malte Herold, Nathan D. Hicks, John D. Gillece, James M. Schupp, Paul Keim, Paul Wilmes

**Affiliations:** 1 Luxembourg Centre for Systems Biomedicine, University of Luxembourg, 7 Avenue des Hauts-Fourneaux, L-4362 Esch-sur-Alzette, Luxembourg; 2 TGen North, 3051 West Shamrell Boulevard, Flagstaff, AZ 86001 USA; 3 Present address: Department of Microbiology, Genomics and the Environment, UMR 7156 UNISTRA – CNRS, Université de Strasbourg, Strasbourg, France; 4 Present address: Saarland University, Building E2 1, 66123 Saarbrücken, Germany

**Keywords:** Genome assembly, Genomic features, Lipid metabolism, Metatranscriptomics, Poly-hydroxyalkanoate, Wastewater treatement plant

## Abstract

The Gram-negative beta-proteobacterium *Zoogloea* sp. LCSB751 (LMG 29444) was newly isolated from foaming activated sludge of a municipal wastewater treatment plant. Here, we describe its draft genome sequence and annotation together with a general physiological and genomic analysis, as the first sequenced representative of the *Zoogloea* genus. Moreover, *Zoogloea* sp. gene expression in its environment is described using metatranscriptomic data obtained from the same treatment plant. The presented genomic and transcriptomic information demonstrate a pronounced capacity of this genus to synthesize poly-β-hydroxyalkanoate within wastewater.

**Electronic supplementary material:**

The online version of this article (10.1186/s40793-017-0274-y) contains supplementary material, which is available to authorized users.

## Introduction

10.1601/nm.2061 spp. are chemoorganotrophic bacteria often found in organically enriched aquatic environments and are known to be able to accumulate intracellular granules of poly-β-hydroxyalkanoate [1]. The combination of these two characteristics renders this genus particulary interesting from the perspective of high-value resource production from wastewater [2, 3]. In particular, PHA may be used to synthesize biodegradable bioplastics or chemically transformed into the biofuel hydroxybutyrate methyl ester [2].

The genus name 10.1601/nm.2061 is derived from the Greek term; meaning ‘animal glue’, which refers to a phenotypic trait that was previously used to differentiate between 10.1601/nm.2061 species and other metabolically similar bacteria [1]. The polysaccharides making up this “zoogloeal matrix” have been proposed to act as a matrix for the adsorption of heavy metals [4].

To date, no genome sequence exists for any of the representative strains of the five presently recognised 10.1601/nm.2061 species and thus, limited information is available with regards to the genomic potential of the genus. Here we report the genome of a newly isolated 10.1601/nm.2061 sp. strain as a representative of the genus, with a focus on its biotechnological potential in particular for the production of biodiesel or bioplastics. Accordingly, we studied the 10.1601/nm.2061 core metabolism of the genus, particularly on the lipid accumulating properties of 10.1601/nm.2061 sp. LCSB751. Moreover, we integrate metatranscriptomic sequencing data to resolve gene expression of this genus in situ [5, 6]. Finally, we also analyze the clustered regularly interspaced palindromic repeats mediated defence mechanisms of 10.1601/nm.2061 sp. LCSB751 to infer putatively associated bacteriophages [7].

## Organism information

### Classification and features

10.1601/nm.2061 sp. LCSB751 was isolated from an activated sludge sample collected from the surface of the first anoxic tank of the Schifflange communal wastewater treatment plant, Schifflange, Luxembourg (49°30′48.29′′N; 6°1′4.53′′E) on 12 October 2011. The activated sludge sample was processed by serial dilution with sterile physiological water to a factor of 10^4^ and the biomass was then cultivated on solid MSV peptone medium [8] at 20 °C and under anoxic conditions (less than 100 ppm oxygen). Single colonies were iteratively re-plated until a pure culture was obtained. The newly isolated 10.1601/nm.2061 sp. LCSB751 was cryopreserved in 10% glycerol at −80 °C.

**Fig. 1:**
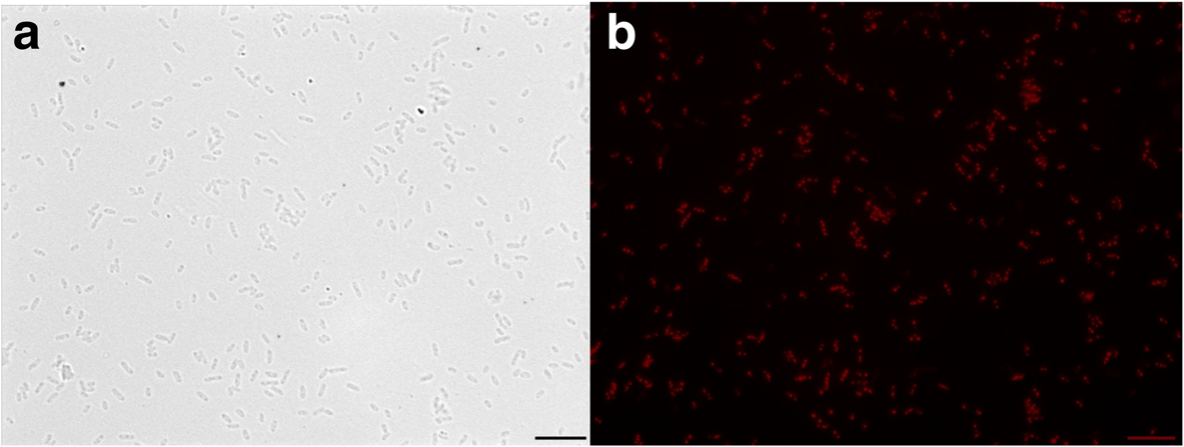
Photomicrograph of 10.1601/nm.2061 sp. strain LCSB751. **a**: bright field of anaerobically grown colonies, Nile Red stained after heat fixation; **b**: same field observed with epifluorescence using an excitation light from a Xenon arc lamp. The beam was passed through an Optoscan monochromator (Cairn Research, Kent, UK) with 550/20 nm selected band pass. Emitted light was reflected through a 620/60 nm bandpass filter with a 565 dichroic connected to a cooled CCD camera (QImaging, Exi Blue). The images were taken using an inverted microscope (Nikon Ti) equipped with a 60× oil immersion Nikon Apo-Plan lambda objective (1.4 N.A) and an intermediate magnification of 1.5×. The scale represents 10 μm. All imaging data were collected and analysed using the OptoMorph (Cairn Research, Kent, UK) and ImageJ

10.1601/nm.2061 sp. LCSB751 is a facultative anaerobe as it was found to also grow aerobically at 20 °C - 25 °C with agitation in the following liquid media: R2A [9], MSV A + B [8] or Slijkhuis A [10]. Cell clumps were observed in all tested culture conditions. When grown on R2A agar or on MSV peptone agar at 25 °C under aerobic conditions, 10.1601/nm.2061 sp. LCSB751 colonies were initially punctiform and after three days, they were white, circular and raised with entire edges. The morphology of cells derived from these growth conditions indicates that these are short rod-shaped bacteria (Fig. 1a). The Gram-staining was negative which is in accordance with previously described isolates of 10.1601/nm.2061 spp. [11, 12] (Table 1).

**Table 1:** Classification and general features of *Zoogloea* sp. strain LCSB751 according to the MIGS recommendation [18]

MIGS ID	Property	Term	Evidence code^a^
	Classification	Domain *Bacteria*	TAS [34]
		Phylum *Proteobacterium*	TAS [35]
		Class *Betaproteobacterium*	TAS [36]
		Order *Rhodocyclales*	TAS [13]
		Family *Rhodocyclaceae*	TAS [13]
		Genus *Zoogloea*	IDA
		Species Unknown	IDA
		Strain: LCSB751	
	Gram stain	Negative	TAS [1]
	Cell shape	Rod	TAS [1]
	Motility	Motile	TAS [1]
	Sporulation	Not reported	NAS
	Temperature range	5–40 °C	TAS [11, 13, 14]
	Optimum temperature	25–30 °C	TAS [11, 13]
	pH range; Optimum	6.0–9.0; 6.5–7.5	TAS [11, 13]
MIGS-6	Habitat	Activated sludge	IDA
MIGS-6.3	Salinity	Inhibited at 0.5% NaCl (*w*/*v*)	TAS [14]
MIGS-22	Oxygen requirement	facultative anaerobe	IDA
MIGS-15	Biotic relationship	free-living	IDA
MIGS-14	Pathogenicity	non-pathogen	NAS
MIGS-4	Geographic location	Luxembourg	IDA
MIGS-5	Sample collection	2011	IDA
MIGS-4.1	Latitude	49°30′48.29′′N;	IDA
MIGS-4.2	Longitude	6°1′4.53′′E	IDA
MIGS-4.4	Altitude	275 m	IDA

^a^Evidence codes - *IDA* Inferred from Direct Assay, *TAS* Traceable Author Statement (i.e., a direct report exists in the literature), *NAS* Non-traceable Author Statement (i.e., not directly observed for the living, isolated sample, but based on a generally accepted property for the species, or anecdotal evidence). These evidence codes are from the Gene Ontology project [37]

**Fig. 2:**
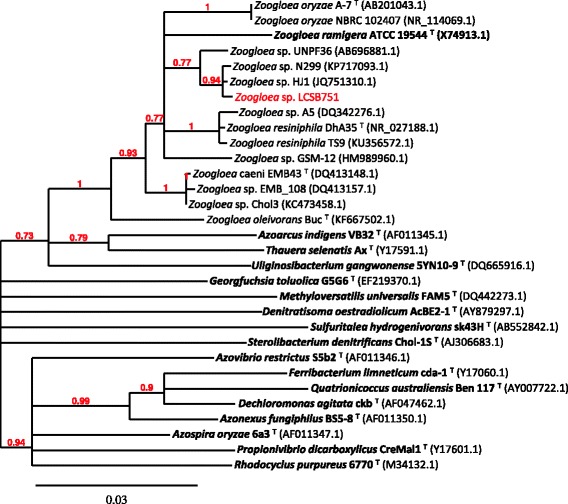
Phylogenetic tree based on 16S rRNA gene sequences. The type species strains of every species of the 10.1601/nm.2016 family were used (in bold) as well as all the type strains of the genus 10.1601/nm.2061, according to the List of Prokaryotic names with Standing in Nomenclature (LPSN; http://www.bacterio.net). Whole genome GenBank IDs are provided in brackets. The 16S rRNA sequences were aligned using ClustalW, the alignment was curated using Gblocks conserving 81% of the initial positions and the phylogeny was computed with BioNJ using 100 bootstraps and the default (K2P) substitution model, using the pipeline Phylogeny.fr [38]

Phylogenetic analysis based on 16S rRNA gene sequences confirmed that strain LCSB751 belongs to the 10.1601/nm.2061 genus of the beta-proteobacterial class (Table 1). However, this strain formed a distinct phyletic linage from the five recognized species of 10.1601/nm.2061, that are represented by the type strains 10.1601/nm.14144 EMB43^T^ [13], 10.1601/nm.26252 Buc^T^ [11], *Z. oryzea* A-7^T^ [14], 10.1601/nm.2062 Itzigsohn 1868 10.1601/strainfinder?urlappend=%3Fid%3DATCC+19544 ^T^ [15] and 10.1601/nm.2063 DhA-35^T^ [16, 17] (Fig. 2).

#### Extended feature descriptions

The capacity of 10.1601/nm.2061 sp. LCSB751 to accumulate intracellular granules of lipids was tested using the dye Nile Red as described by Roume, Heintz-Buschart et al. [5]. Figure 1b shows the Nile Red positive phenotype of the described strain.

Additionally, the growth characteristics of the strain 10.1601/nm.2061 sp. LCSB751 were determined aerobically and at 25 °C with agitation in 3 different liquid media. Its generation time was the longest in Slijkhuis A medium with the highest biomass production. MSV A + B allowed a generation time of 4 h 30 min but lead to a poor biomass production as demonstrated by the low maximal optical density at 600 nm (OD_600_) of 0.21_._ The tested liquid medium which allowed the fastest growth for 10.1601/nm.2061 sp. LCSB751 was R2A while the biomass production was close to those observed for Slijkhuis A (Table 2).

**Table 2:** Generation time, growth rate and maximum growth of 10.1601/nm.2061 sp. LCSB751 under different aerobic culture conditions

Medium	Generation time ± standard deviation^a^	Growth rate (min^−1^)	Maximum OD_600_^b^
R2A	1 h 54 min ± 3 min	0.0058	0.46
MSV A + B	4 h 30 min ± 53 min	0.0026	0.21
Slijkhuis A	10 h 42 min ± 1 h 51min	0.0011	0.73

^a^Values are an average of independent triplicate experiments

^b^OD_600_ stands for optical density measured at 600 nm with the spectrometer “Biochrom WPA CO 8000 Cell Density Meter” using BRAND disposable semi-micro UV cuvettes of 12.5 × 12.5 × 45 mm

## Genome sequencing information

### Genome project history

Overall, 140 pure bacterial isolates were obtained from a single activated sludge sample, and screened for lipid inclusions using the Nile Red fluorescent dye. The genomes of 85 Nile Red-positive isolates were sequenced, of which isolate LCSB065 has already been published [5]. In particular, the genome of 10.1601/nm.2061 sp. LCSB751 was analyzed to obtain information about the functional potential of this genus, which has no publically available representative genome sequence, but also based on its particular phylogenetic position and to acquire knowledge on the genes related to lipid accumulation. The permanent draft genome sequence of this strain is available on NCBI with the GenBank accession number MWUM00000000 (BioSample: SAMN06480675). Table 3 summarizes the project information according to the MIGS compliance [18].

**Table 3:** Project information

MIGS ID	Property	Term
MIGS 31	Finishing quality	Draft
MIGS-28	Libraries used	Illumina paired-end reads (insert size 30 bp)
MIGS 29	Sequencing platforms	Illumina HiSeq
MIGS 31.2	Fold coverage	150×
MIGS 30	Assemblers	SPAdes (version 3.1.1)
MIGS 32	Gene calling method	RAST server^a^ and Prokka^b^
	Locus Tag	fig|6666666.102999
	Genbank ID	MWUM00000000
	GenBank Date of Release	15 March 2017
	GOLD ID	Gs0128811
	BIOPROJECT	PRJNA230567
MIGS 13	Source Material Identifier	LMG 29444
	Project relevance	Environmental, biodiversity, biotechnological

^a^Gene calling using GLIMMER [27, 39]

^b^Gene calling using Prodigal [26, 40]

### Growth conditions and genomic DNA preparation

10.1601/nm.2061 sp. LCSB751 was grown on MSV peptone agar medium [8] at 20 °C under anoxic conditions. Half of the biomass was scrapped in order to cryopreserve the strain, while the second half was used for DNA extraction using the Power Soil DNA isolation kit (MO BIO, Carlsbad, CA, USA). This cryostock was used to distribute the strain to the Belgian Coordinated Collection of Microorganisms collection center and deposited under number 10.1601/strainfinder?urlappend=%3Fid%3DLMG+29444.

### Genome sequencing and assembly

The purified DNA was sequenced on an Illumina Genome Analyzer IIx as previously described by Roume, Heintz-Buschart and colleagues [5]. Briefly, a paired-end sequencing library with a theoretical insert size of 300 bp was prepared with the AMPure XP/Size Select Buffer Protocol as previously described by Kozarewa & Turner [19], modified to allow for size-selection of fragments using the double solid phase reversible immobilization procedure [20] and sequenced on an Illumina HiSeq with a read length of 100 bp at TGen North (AZ, USA). The resulting 2,638,115 paired-end reads were trimmed of N bases (i.e. minimum phred quality score of 3 and filtered for Illumina TruSeq3 adapters), retaining 2,508,729 (~95%) of paired reads, 129,378 and eight forward- and reverse-singleton reads (i.e. mate pair discarded), respectively. All reads retained (paired-end and singleton reads) after the pre-processing were de novo assembled using SPAdes ver. 3.1.1, using the default *k*mer range and parameters [21].

**Fig. 3:**
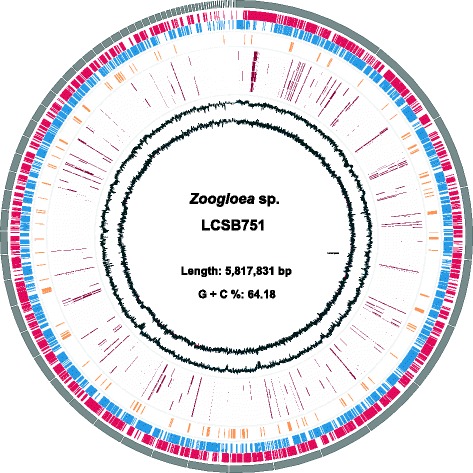
Circular graphical map of the 10.1601/nm.2061 sp. LCSB751 draft genome assembly, annotation and in situ expression. Data shown on the map explained from the outer to inner circles (i-x): i) contigs above 1 kb. Accordingly, all subsequent information contained within inner circles are based on these contigs, including ii) forward strand coding sequences in red (CDS), iii) reverse strand CDS in blue, iv) CDS that are related to lipid accumulation in yellow (forward and reverse strands), v-viii) gene expression in situ based on metatranscriptomic data from four sampling dates (25 January 2011, 11 January 2012, 5 October 2011, and 12 October 2011 [6]) ix) GC-deviation (from overall G + C %) and x) GC-skew, respectively. Graphics were generated using Circos [41]. CDS were predicted and annotated using the RAST server [27]. Metatranscriptomic data from four sampling dates were aligned against the draft genome using BWA [42] and depth of coverage, computed using BEDtools [25] was used as a proxy for expression. Depth of coverage <0.3 were set to zero

The total number of contigs (776), the mean contig length (7497 bp) and the N50 value (180,423 bp) of the draft assembly of 10.1601/nm.2061 sp. LCSB751 (Table 3) indicate a fragmented assembly despite an estimated sequencing depth of ~150× fold coverage, ~100× based on 21-mer frequencies (using KMC2 [22]) and a ~ 120× average depth of coverage based on mapping reads back onto the de novo assembled contigs [23–25]. Assembled contigs above 1 kb are represented in Fig. 3.

### Genome annotation

Gene (i.e. open reading frame) prediction and annotation was carried out on the assembled contigs using Prokka ver. 1.11 [26] and the RAST server [27], both executed using default parameters and databases. Briefly, Prokka predicted a total of 5200 features including 5118 CDS, 3 rRNA, 76 tRNA genes and one tmRNA genes as well as two repeat regions. Similarly, the RAST server predicted a total of 5202 features, of which 5125 represent coding sequences (CDS), 6 rRNA and 71 tRNA genes. The annotation derived from the RAST server was used for most of the genome descriptions and downstream analyses, unless explicitly mentioned. CDS on the forward and reverse strands within contigs above 1 kb are represented in Fig. 3. In addition, the proteins predicted by the RAST server were submitted to i) the WebMGA server [28], ii) the SignalP server v.4.1 [29] and iii) the TMHMM server v.2.0 [30], for COG functional annotation, signal peptides prediction and transmembrane helices prediction, respectively. 5202 of the predicted amino acid sequences were annotated with 13,030 Pfam IDs. Finally, metaCRT [31] was used to predict CRISPR loci and the resulting CRISPR-spacers were submitted to the CRISPRtarget server [32] for the identification of putatively associated bacteriophage sequences.

## Genome properties

The draft genome assembly of 10.1601/nm.2061 sp. LCSB751 consists of 5,817,831 bp with a G + C content of 64.2%, distributed over 776 contigs (773 scaffolds) with an N50 value of 180,423 bp (Table 4), GC-skew and –deviation of contigs above 1 kb are represented in Fig. 3. The raw reads are available via the GenBank nucleotide database under the accession number MWUM00000000, while the assembly and the annotation (IDs 6666666.102999) can be accessed through the RAST server guest account.

**Table 4:** Genome statistics of *Zoogloea* sp. LCSB751

Attribute	Value	% of Total^a^
Genome size (bp)	5,817,831	100.00
DNA coding (bp)^b^	4,966,077	85.36
DNA G + C (bp)	3,733,728	64.18
DNA scaffolds	773	100.00
Total genes	5,202^c^ / 5,200^d^	100.00^c^ / 100.00^d^
Protein coding genes	5,125^c^ / 5,118^d^	98.52^c^ / 98.42^d^
RNA genes	77^c^ / 80^d^	1.48^c^ / 1.54^d^
Pseudo genes	unknown	unknown
Genes in internal clusters	unknown	unknown
Genes with function prediction ^c^	3661	70.38
Genes assigned to COGs	4191	80.56
Genes with Pfam domains	4202	80.78
Genes with signal peptides	505	9.71
Genes with transmembrane helices	1157	22.24
CRISPR repeats	2^d^ / 3^e^	2.85

^a^Total is based on either the size of the genome in base pairs, total number of scaffolds or the total number of genes in the annotated genome

^b^Cumulative length of genes, without considering overlaps

^c^As predicted by RAST server [27]

^d^As predicted by Pokka [26]

^e^As predicted by MetaCRT [31]

The rRNA operon region is assumed to be occurring in multiple copies, because all reads from this region were assembled into a single contig with a higher depth of coverage (~1200×, for RAST server features: fig|6666666.102999.rna.57, fig|6666666.102999.rna.60 and fig|6666666.102999.rna.61) compared to the rest of the genome. All 20 regular amino-acids were covered by tRNA-anticodons. The RAST server and Prokka annotated approximately 22% (1139) and 26% (1329) of the CDS as hypothetical proteins or proteins of unknown function, respectively. The distribution of COG functional categories are reported in Table 5, while subsystem-based functional classification are available via RAST server.

**Table 5:** Number of genes associated with general COG functional categories

Code	Value	%age	Description
J	182	3.50	Translation, ribosomal structure and biogenesis
A	3	0.06	RNA processing and modification
K	342	6.57	Transcription
L	204	3.92	Replication, recombination and repair
B	3	0.06	Chromatin structure and dynamics
D	52	1.00	Cell cycle control, Cell division, chromosome partitioning
V	69	1.33	Defense mechanisms
T	564	10.84	Signal transduction mechanisms
M	252	4.84	Cell wall/membrane biogenesis
N	177	3.40	Cell motility
U	142	2.73	Intracellular trafficking and secretion
O	189	3.63	Posttranslational modification, protein turnover, chaperones
C	362	6.96	Energy production and conversion
G	130	2.50	Carbohydrate transport and metabolism
E	305	5.86	Amino acid transport and metabolism
F	85	1.63	Nucleotide transport and metabolism
H	185	3.56	Coenzyme transport and metabolism
I	202	3.88	Lipid transport and metabolism
P	283	5.44	Inorganic ion transport and metabolism
Q	126	2.42	Secondary metabolites biosynthesis, transport and catabolism
R	520	10.00	General function prediction only
S	351	6.75	Function unknown
–	1011	19.43	Not in COGs

Percentage (%) is based on the total number of protein coding genes in the genome

## Insights from the genome sequence

### Genome-based inference of the central metabolism

The genome of 10.1601/nm.2061 sp. LCSB751 is predicted to encode for all the genes required for a complete TCA cycle, but is missing some or the complete set of genes for the EMP pathway, the pentose phosphate pathway and the Entner-Doudoroff pathway.

A periplasmic nitrate reductase as well as a nitrite reductase were identified, suggesting complete reduction of nitrate to ammonia by 10.1601/nm.2061 sp. LCSB751. Furthermore, a complete set of *nif* genes involved in nitrogen fixation were also encoded in the genome.

Genes for a complete electron transport chain were predicted as well as an alternative RNF complex [33].

The genome of 10.1601/nm.2061 sp. LCSB751 also encodes numerous genes for flagella synthesis and assembly, suggesting a motile lifestyle. Furthermore, the strain is predicted to be prototroph for all amino acids, nucleotides and vitamins B_2_, B_6_, B_9_, H, and is missing a single gene for the synthesis of B_12_.

Additionally, the catechol 2,3-dioxygenase that has been studied in 10.1601/nm.26252, was found to be encoded by the genome of 10.1601/nm.2061 sp. LCSB751 [11].

### Lipid metabolism

The genome of 10.1601/nm.2061 sp. LCSB751 was further analysed with a focus on genes related to lipid metabolism, to better understand the lipid accumulation properties of 10.1601/nm.2061 spp. With 202 genes annotated with COG functional category I “Lipid transport and metabolism”, more than 3.8% of the genome of 10.1601/nm.2061 sp. LCSB751 is potentially dedicated to lipid metabolism (Table 5 and Fig. 3). Using the SEED subsystem feature, similar results were obtained with 194 genes (3.8%) classified in the “Fatty acids, lipids and Isoprenoids” subsystem (Table 6).

**Table 6:** Gene abundance and frequency related to the lipid metabolism of 10.1601/nm.2061 sp. LCSB751

Subsystem	Subsystem feature count	Subsystem feature (%)
**Fatty acids, lipids and isoprenoids**	**194**	**100**
**Phospholipids**	**30**	**15.46**
Cardiolipin synthesis	2	6.67
Glycerolipid and glycerophospholipid metabolism in bacteria	28	93.33
**Triacylglycerols**	**3**	**1.55**
Triacylglycerol metabolism	3	100
**Fatty acids**	**71**	**36.60**
Fatty acid biosynthesis FASII	30	42.25
Fatty acid metabolism cluster	41	57.75
**Fatty acids, lipids and isoprenoids - no subcategory**	**56**	**28.87**
Polyhydroxybutyrate metabolism	56	100
**Isoprenoids**	**34**	**17.53**
Isoprenoids for quinones	5	14.71
Isoprenoid biosynthesis	18	52.94
Polyprenyl diphosphate biosynthesis	4	11.76
Nonmevalonate branch of isoprenoid Biosynthesis	7	20.59

The different categories (in **bold**) and subcategories of the subsystem “Fatty acids, lipids and isoprenoid” are represented

Specifically, a complete set of predicted genes necessary for the synthesis, polymerisation and depolymerisation of PHA [2] was found as well as the genes of the MEP/DOXP pathway for terpenoid synthesis. However, the gene necessary to convert diacylglycerol in triacylglycerol or fatty alcohol in wax ester was not predicted, suggesting that PHA granules are the only lipid bodies accumulated in 10.1601/nm.2061 sp. LCSB751.

### In situ gene expression

While genomic data provides information about the genetic potential of 10.1601/nm.2061 sp. LCSB751, it is possible to study expressed functions of the 10.1601/nm.2061 population in situ by using metatranscriptomic data derived from the biological wastewater treatment plant this strain originated from. Metatranscriptomic data derived from samples collected at four distinct time points (25 January 2011, 11 January 2012, 5 October 2011, and 12 October 2011), as studied by Muller and collaborators [6] was used herein. Genes with an average depth of coverage equal or higher than 0.3 were considered as expressed by mapping the rRNA-depleted transcripts on the genome of 10.1601/nm.2061 sp. LCSB751. 259, 312, 269 and 330 genes, respectively, were expressed, with 160 of them being expressed at all four time points (Fig. 3 and Additional file 1: Table S1). For the vast majority, (4732 genes), no transcripts were detected, which can be explained by the low population size of 10.1601/nm.2061 sp. in situ. This was estimated by phylogenetic marker gene (16S rRNA) amplicon sequencing on the sample collected on 25 January 2011 (data from [6]), for which the 10.1601/nm.2061 sp. population size was estimated at 0.1%. Similarly, metagenomic data from all the samples further support the low abundance of this strain in situ (Additional file 1: Table S2).

Nitrate reductase encoding genes (specifically the periplasmic nitrate reductase; NapA) were found to be expressed in all the four time points, while nitrite reductase or nitrogen fixation genes were sporadically expressed in those four time points. Interestingly, at least one copy of the acetoacetyl-CoA reductase and of the polyhydroxyalkanoic acid synthase were found to be expressed at each time point, possibly suggesting PHA accumulation by the population of 10.1601/nm.2061 sp. in this environment. Additionally, the third most expressed gene of 10.1601/nm.2061 sp. in this environment is a “granule associated protein (phasin)” typically known to be associated with PHA granules.

### CRISPR-*Cas* system and putative bacteriophages

A total of three CRISPR loci were detected with metaCRT, accompanied by six CRISPR-associated (*cas*) genes. Five of the predicted *cas* genes occur consecutively, within the same contig and all of the predicted *cas* genes occur adjacent to a CRISPR locus [7]. Two of CRISPR repeats types were 37 bp in length (sequence: GTTTCAATCCACGTCCGTTATTGCTAACGGACGAATC; GTGGCACTCGCTCCGAAGGGAGCGACTTCGTTGAAGC) while one of them is 32 bp (sequence: CACTCGCTCCGGAGGGAGCGACTTCGTTGAAG). These CRISPRs contain 175, 51 and 11 spacers, respectively, ranging from lengths of 33 to 46 bp. A total of 77 matches were found when searching the spacers against the ACLAME phage/viral/plasmid gene database, NCBI phage and NCBI virus databases using the CRISPRtarget tool [32]. 51 of the spacers match to bacteriophages, 6 to viruses, 11 to genes within plasmids and six to genes within prophages (Additional file 1: Table S3). Based on the available metatranscriptomic data, minute to no expression of the *cas* genes was observed, while the detected CRISPR regions were not covered by the metatranscriptomic data (Additional file 1: Table S1). This is likely due to the overall low abundance of this species in situ (Additional file 1: Table S2).

## Conclusions

We describe the first draft genome of a strain potentially belonging to a novel species within the genus 10.1601/nm.2061. The genetic inventory of 10.1601/nm.2061 sp. LCSB751 makes it of particular interest for future wastewater treatment strategies based around the comprehensive reclamation of nutrients and chemical energy-rich biomolecules around the concept of a “wastewater biorefinery column” [3] as well as for industrial biotechnological applications. Future comparative genomics studies would allow the scientific community to further confirm if the reported genomic repertoire is indeed typical of this genus. Using metatranscriptomic data, we further show that 10.1601/nm.2061 sp. populations are active in the studied wastewater treatment plant despite being low in abundance and likely accumulate PHA in situ.

## Additional file

Additional file 1: Table S1. Metatranscriptomic coverage for the predicted features of *Zoogloea* sp. LCSB751. **Table S2.** Metagenomic coverage for the assembly contigs of *Zoogloea* sp. LCSB751. **Table S3.** *Zoogloea* sp. LCSB751 CRISPR spacer complements (protospacer) as per reported by CRISPRTarget [32]. (XLSX 182 kb)

## Abbreviations

COG: Clusters of Orthologous Groups
CRISPR: Clustered regularly interspaced palindromic repeats
PHA: Poly-β-hydroxyalkanoate
*Cas*: CRISPR-associated
